# Consortium of Indigenous Fecal Bacteria in the Treatment of Metabolic Syndrome

**DOI:** 10.3390/microorganisms10081574

**Published:** 2022-08-05

**Authors:** Elena Ermolenko, Marina Kotyleva, Anna Kotrova, Sergey Tichonov, Nadezhda Lavrenova, Lyubov Voropaeva, Yulia Topalova, Alena Karaseva, Daniil Azarov, Konstantin Ermolenko, Dmitrii Druzhininskii, Alexander Dmitriev, Alexander Shishkin, Alexander Suvorov

**Affiliations:** 1 Federal State Budgetary Institution “Institute of Experimental Medicine”, 197376 Saint-Petersburg, Russia; 2 Medical Faculty, Saint-Petersburg State University, 199034 Saint-Petersburg, Russia; 3 Department of Internal Medicine, Clinical Pharmacology and Nephrology, North-Western State Medical University named after I.I. Mechnikov, 195067 Saint-Petersburg, Russia; 4 Pediatric Research and Clinical Center for Infectious Diseases, 197022 Saint-Petersburg, Russia; 5 Almazov National Medical Research Centre, 197341 Saint-Petersburg, Russia

**Keywords:** *Lactobacillus* spp., *Bifidobacterium* spp., *Bacteroides fragilis*, indigenous consortium, obesity, eating disorders, dyslipidemia, autoprobiotics

## Abstract

The features of gut microbiota in metabolic syndrome (MS) and ways to correct it using autoprobiotics, based on indigenous bacteria obtained from fecal samples of the host, remain unexplored. The aim of the study was to investigate the effectiveness of an indigenous consortium (IC) of fecal bacteria in treatment of patients with MS. The study was carried out on 36 patients with MS, manifested with abdominal obesity, eating disorders, dyslipidemia, and hypertension. The control group was formed by 20 healthy volunteers. Samples of IC and gut microbiota content were examined by qPCR and metagenome (16S rRNA) analysis before and after therapy. The decrease in anthropometric parameters of obesity, liver enzyme level correction, reduction in C reactive protein and triglyceride concentrations were revealed after IC usage. The decrease in genera *Bifidobacterium*, *Enterobacter*, *Paraprevotella*, and *Prevotella*, as well as an increase in *Bacteroides fragilis* and *Oscillospira* spp. populations were shown after consumption of IC. A negative correlation between the quantity of *B. fragilis* and the anthropometric parameters of obesity (r = −0.48) and C reactive protein level (r = −0.36) in serum was established. Thus, IC can be considered as a potential functional personified product for the therapy of MS.

## 1. Introduction

Metabolic syndrome (MS), represented by visceral obesity with dyslipidemia, hyperglycemia, and hypertension, has become one of the major public-health challenges worldwide [1]. The epidemiological and clinical studies data indicate that eating disorders, sedentary lifestyle, and a fat-rich diet with a low fiber content are the main modifiable factors in the treatment of various variants of MS. [2]. One of its developmental pathogenetic factors is gut microorganism disorders [3]. It was shown that dysbiosis could be involved in the MS development by relating the oxidative stress with an inflammatory process [4]. The methods of gut microbiota correction such as probiotics, prebiotics, nutraceuticals, and fecal microbiota transplantation (FMT) may represent effective forms for improvement in MS course [5,6].

Probiotic lactobacilli were mainly used in the treatment of MS [7], which promote the changes in intestinal microbiome, normalization of the lipid and carbohydrate metabolism, and weight reduction. However, due to the lack of biocompatibility between the host organism and its microbiota to the administered probiotics, they are eliminated rapidly from the organism, and longer treatment periods are needed [8]. In some studies, dysbiosis condition could be corrected with the use of autoprobiotics, the indigenous beneficial host bacteria which have a high potential for overcoming these problems [9].

Autoprobiotics are indigenous representatives of the normal microbiota of host organisms (lactobacilli, enterococci, bifidobacteria and their mixture, isolated from the organism and orally consumed by the host after cultivation in the same concentrations as probiotics). Their indisputable advantages are immunological tolerance, compatibility with the host microbiota and the metabolic status, allowing autoprobiotics to persist in the body for a long time [8]. The efficacy of mono-strain autoprobiotics has been proven in an antibiotic-associated intestinal dysbiosis experimental model [9,10] and in treatment of irritable bowel syndrome and pneumonia [11]. However, the introduction of single components of intestinal microbiota cannot recreate the complete microbiota, characterized by a high biodiversity and the presence of its obligate representatives. Microbial fecal transplantation (FMT) is preferable in this case [12]. However, FMT has logistical challenges and is associated with several problems: infections, undesirable outcomes (e.g., increased risk of microbiota-associated diseases), and so called ‘yuck’ factor [13]. Efforts are also under way to create artificial microbiota using fecal samples as source of own or donor bacteria. To date, no attempts have been made to use new generation of autoprobiotics, an indigenous consortium based on own fecal bacteria for the treatment of somatic diseases. Previously, its effectiveness was tested on an experimental model of dysbiosis [9].

**The aim** of the study was to investigate the effectiveness of an IC of fecal bacteria in treatment of patients with MS analyzing the anthropometric parameters, eating behavior, metabolism, inflammatory markers, and microbiome restoration.

## 2. Materials and Methods

### 2.1. Characteristics of the Study Participants

The study participants were patients with MS, manifested in abdominal obesity, dyslipidemia, and arterial hypertension. Patients were followed up in the clinics of the Saint-Petersburg State University and North-Western State Medical University named after I.I. Mechnikov. All patients signed an informed consent. The inclusion criteria in the group of MS were: age of patients from 25 to 60 years (Figure S1), abdominal obesity, assessed by waist circumference (WC) of more than 80 cm in women and more than 94 cm in men, with arterial hypertension (blood pressure ≥ 130/85 mmHg), and decreased high-density lipoprotein cholesterol (HDL cholesterol < 1, 0 mmol/L in men; <1.2 mmol/L in women) or increased levels of low-density lipoprotein cholesterol (LDL cholesterol > 3.0 mmol/L) or increased triglyceride levels (TG ≥ 1.7 mmol/L).

Exclusion criteria for patients from the study were thyrotoxicosis, hypothyroidism, type 1 diabetes, decompensated type 2 diabetes, oncological diseases, organic intestinal pathology, acute intestinal infections in the past six months, taking antibacterial drugs or probiotics in the last 6 months. Healthy volunteers aged 25–60 with normal body weight (BMI = 18.5–24.9 kg/m^2^), with BP ≤ 120–129/80–84 mmHg, with normal lipid profile and normal fasting blood glucose formed the control group. The study was carried out on 36 patients with MS and 20 healthy volunteers (HV). The subjects included 13 men and 23 women (Figure S1a). The mean age in the group was 47.0 ± 2.8 (20–74) years (Figure S1b). The control group was formed by HV of similar age and gender, and same proportion of male/female subjects as in the MS group.

### 2.2. Ethics Approval

All studies were performed with the informed consent of the subjects and in accordance with the Helsinki Declaration of the World Medical Association “Ethical Principles for Medical Research Involving Human Subjects” with amendments from 2013 [14] and “Rules of Clinical Practice in the Russian Federation” approved by the Order of the Ministry of Health of the Russian Federation 19.06.2003 No. 266.

### 2.3. Study Design

The study design is presented in Table S1 (Supplementary Materials). The experimental group of patients received personalized therapy with IC as a personified functional food product 2 times a day for 10 days. The control group received a similar product without probiotic supplements in a daily dose of 50 mL of fermented soy protein product 2 times a day for 10 days. Before starting therapy, fecal samples were collected from patients to obtain IC. Fecal and blood samples were taken before starting therapy and two weeks after autoprobiotic therapy to study the composition of the intestinal microbiota and biochemical parameters of blood serum. Before and after therapy, patients with MS were examined anthropometrically and surveyed to identify the features of eating behavior. During the experiment, the changes in the main clinical symptoms of MS were analyzed after administration of IC. The patients were examined by a gastroenterologist before and after therapy and filled out gastroenterological diaries daily.

All laboratory tests were carried out on the day of the blood collection. The blood samples were obtained by the peripheral vein puncture and collected in vacuum tubes. Fecal matter was collected from patients before and after therapy, stored in a freezer at 80 °C, and used for IC creation metagenome study.

### 2.4. Preparation of Indigenous Consortium

A functional personified food product based on IC was prepared in the base of own patients’ fecal samples as described in RF patent No. 2,734,896 C2.

Samples of 50 mg feces (previously frozen −80 °C) were transferred into 10 mL of thioglycol medium (PDB, Conda-Pronadisa Laboratories, Madrid, Spain) with addition of 250 μL of 40% sterile glucose solution and 5 μL of vicasol (Joint Stock Company DALKHIMPHARM, Khabarovsk, Russia), then mixed in the lower part of the test tube and throughout the volume of the medium. The tubes were placed in a thermostat for cultivation at a temperature of 37 °C for 6 days.

Inoculation of IC occurred in 10 mL of medium protein-vitamin soy product Supro Plus 2640 (Monsanto company, Missouri, United States, concentration 40 g/L), 1 mL of the consortium was centrifuged (MiniSpin centrifuge (Eppendorf)) for 15 min at 3.5 thousand rpm, then supernatant was discarded and 10 mL of Supro Plus 2640 were added to the sediment and were mixed. The samples were incubated at a temperature of 37 °C under anaerobic conditions for 1 day. Then, 7 mL of the fermented product were added to the 1 L of Supro 2640 and the bottle was incubated for 48–56 h at 37 °C.

Final personified IC products were tested by bacteriological method and qPCR. Exclusion criterion for admission was the presence of pathogenic and opportunistic bacteria.

### 2.5. Intestinal Microbiota Study

Intestinal microbiota was studied bacteriologically by the quantitative polymerase chain reaction (qPCR) and by metagenome analysis (16S rRNA).

### 2.6. Bacteriological Study of Indigenous Consortium

Consortium samples were diluted (diluted 100–10,000 times). The following differential diagnostic media were seeded: saline mannitol agar, Uroselect (Himedya, Thane West, Maharashtra, India). Identification of the grown bacteria was carried out with using MALDI-TOF mass-spectrometry with biotype Bruker Daltonics, Bremen, Germany.

### 2.7. Quantitative Polymerase Chain Reaction

Samples of IC and feces of patients were examined before and after the introduction of the IC by qPCR using the kit Colonoflor (AlphaLab, Saint-Petersburg, Russia) on Mini-Opticon (Applied Biosystems, 850 Lincoln Centre Drive, Foster City, CA, USA). The quantity content of *Acinetobacter* spp., *Citrobacter* spp., *Escherichia coli* and enteropathogenic *E. coli*, *Proteus* spp., *Lactobacillus* spp., *Bifidobacterium* spp., *Bacteroides thetaiotaomicron*, *Bacteroides fragilis* group, *Clostridioides difficile*, *Clostridium perfringens*, *Enterococcus* spp., *Faecalibacterium prausnitzii*, *Fusobacterium nucleatum*, *Parvimonas micra*, *Roseburia inilinivorans*, and *Akkermansia muciniphila* content in intestinal microbiota was studied.

### 2.8. Microbiome (16S rRNA) Study

The IC personified products, fecal samples before and after therapy were investigated by 16S rRNA gene-based metagenomics analysis using a previously described approach [9]. DNA were isolated from feces using the kit «DNA Express bio» Alkor Bio (Saint Petersburg, Russia). Metagenome study was used for the analysis of Libraries of hypervariable regions V3 and V4 of the 16S RNA genes using MiSeq (Illumina, San Diego, CA, USA). The standard method recommended by Illumina based on employing two rounds of PCR was used to prepare the libraries.

Fastqc (http://www.bioinformatics.babraham.ac.uk/projects/fastqc, accessed on 8 January 2019) was used to evaluate the quality of raw reads. CD-HIT-OTU-Miseq [15] was used for OTU retrieval. OTUs were annotated using Greengenes database version 13.5 [16].

### 2.9. Biochemical Analysis

The biochemical study of serum blood samples was performed automatically by Abbot ARCHITECT si 8200 (333 Fiske Street, Holliston, MA, USA). The following parameters were studied: total cholesterol (TC), triglycerides (TG), high-density lipoproteins (HDL), low-density lipoproteins (LDL), alanine aminotransferase (ALT), aspartate aminotransferase (AST), glucose, C-reactive protein (CRP) concentrations in blood serum were determined.

### 2.10. Physical Examination

Patients of both groups were examined by a physician to assess eligibility for inclusion in the study. Waist circumference, height, weight, and blood pressure were measured. Body mass index was calculated to evaluate the severity of overweight.

### 2.11. Eating Behavior

The patients’ eating behaviors were assessed according to the Dutch DEBQ Eating Behavior Questionnaire [17]. Patients completed a 33-question questionnaire on their own prior to taking the IC. According to the results of the survey, the patient could be assigned to one of three types of eating behavior: restrictive, emotional, or external. Evaluation of differences in eating behavior in the compared groups was carried out.

### 2.12. Gastro Diary

During 10 days of taking IC, patients kept gastro diaries, which included the following indicators: stool frequency, Bristol Stool Form Scale, the presence and severity of abdominal pain and tympanites (0—no pain, tympanites; 1—moderate pain, tympanites; 2—severe pain, tympanites) [18].

### 2.13. Statistical Analysis

Statistical analysis was performed using the software package Statistica 8.0. (StatSoft, Tulsa, OK, USA). Differences between the groups were obtained using Kruskal–Wallis tests and ANOVA with post-hoc HSD test for unequal *n*, *p* < 0.05 was considered significant. The normality of the data distribution was determined using the Kolmogorov–Smirnov criterion. The nonparametric criteria were used on this basis. Statistic data processing was performed using the software package IBM SPSS Statistics-22 (IBM, Armonk, NY, USA) and Statistica-10 (Statsoft, Tulsa, OK, USA). The presence of statistically significant differences among groups were determined using the Mann–Whitney U-criterion, adjusted for multiple comparisons by the Benjamini–Hochberg method. Wilcoxon’s *t*-test was also used for paired samples. Comparative analysis was conducted using the a posteriori test honestly significant difference (HSD) for unequal N in the Statistica-8 software. A search for correlations between the studied parameters was performed using Spearman’s test using the software package Statistica 10.0. (StatSoft, Tulsa, OK, USA). χ2 tests with Yates correction were used. Differences in *p* < 0.05 were considered significant.

## 3. Results

### 3.1. Indigenous Consortium Content

The IC were prepared from 44 samples. However, after their bacteriological examination and with the help of qPCR, elevated levels of *Clostridium difficille*, *C. perfringens*, *Proteus* spp., *Enterobacter* spp., and *Candida albicans* were detected in 8 samples. These samples were regarded as contaminated and dangerous and were not used for clinical studies. A comparative study of the IC and fecal samples content was also performed using qPCR and metagenome analysis.

#### 3.1.1. qPCR

**Figure 1:**
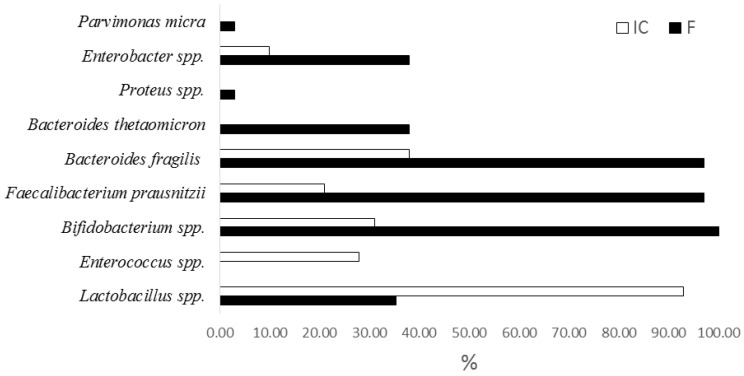
Frequency of different taxa identification in the composition of fecal samples and the indigenous consortium by qPCR study. Notes: *Lactobacillus* spp. > 5 lg CFU/g; *Enetrococcus* > 5 lg CFU/g; F—fecal samples; IC—indigenous consortium. Only statistically reliable results are presented. *p* < 0.05 were determined by χ2 test with Yates correction.

**Figure 2:**
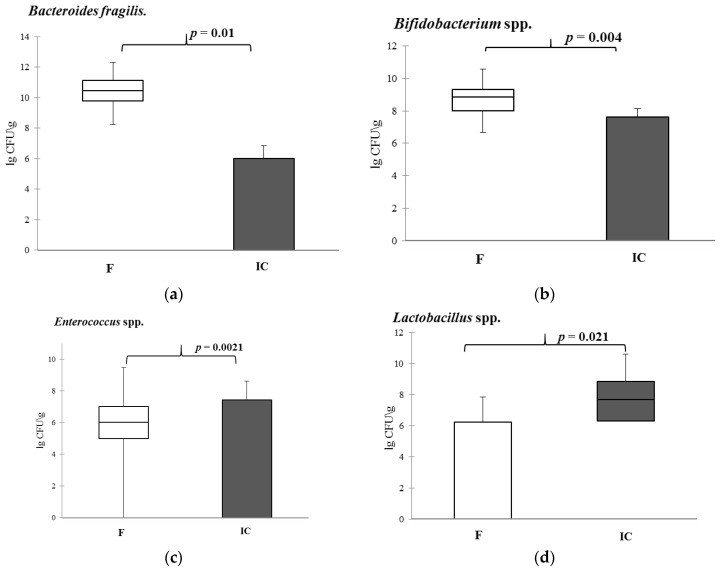
Quantity content of *Bifidobacterium* (**a**), *Bacteroides fragilis* (**b**), *Enterococcus* (**c**) and *Lactobacillus* (**d**) genera in the fecal samples and indigenous consortiums. Notes: F—fecal samples; IC—indigenous consortium. Results are presented as median (25%; 75%).

It was shown that the quantity of all controlled marker bacteria is reduced, but to varying frequencies and degrees (Figure 1, Table S2, Supplementary Materials). When analyzing the frequency of detection of individual taxa, it was found that after prolonged cultivation of fecal samples, the frequency of detection of conditionally pathogenic bacteria disappeared or decreased (*Parvimonas micra*, *Proteus* spp., *Enterobacter* spp. (Figure 2)). At the same time, the proportion of samples containing enterococci and lactobacilli in an amount of more than 5 lg CFU/g (more often χ2, *p* < 0.05) was increased in IC. In a number of samples, despite the absence of strictly anaerobic cultivation conditions in the consortium, representatives of genera *Faecalibacterium*, *Bifidobacterium*, and *Bacteroides* remained in a fairly high percentage of samples (in 20–40%).

It should be noted that the decrease in the quantitative content of *Bifidobacterium* spp., *Enterococcus* spp., and *Bacteroides* spp. was significantly less than in other bacterial genera studied by qPCR (Figure 2a–c; Table S3 in Supplementary Materials. In addition, a sharp increase in lactobacilli attracted attention (Figure 2d, Table S2).

#### 3.1.2. Metagenome Study 16S rRNA

The difference was noted in the alpha-diversity of MS fecal microbiome and IC, which was predictably decreased after cultivation of fecal samples (Figure S2, Supplementary Materials). Despite the fact that biological diversity was significantly reduced, interesting data were obtained using the qPCR method when analyzing the frequency of detection of individual bacterial genera. In total, 72 genera of bacteria were identified in the feces of patients with MS. After culturing fecal samples, the representation of identified genera decreased to 19. Table 1 shows the proportion of samples in which individual genera were detected in feces and “disappeared” (representation less than <0.0001) or decreased after cultivation. Thus, elimination of, or a decrease in, the identification frequency of the following opportunistic bacterial genera was revealed: *Campylobacter*, *Citrobacter*, *Desulfovibrio*, *Haemophilus*, *Paraprevotella*, *Prevotella*, *Streptococcus*, *Granulicatella*, *Burkholderia*, *Pseudomonas*, and *Veillonella*.

In the selective growing conditions of the anaerobic consortium, *Lactobacillus* develops in significant quantities (86%). At the same time, the occurrence or detection frequency of beneficial lactic acid bacteria, belonging to the genera *Enterococcus*, *Lactobacillus*, and *Pediococcus* was increased. The exception of this trend were bacteria, on the basis of which probiotics were prepared, including for the treatment of MS: *Bifidobacterium* spp. and *Akkermansia* spp. The detection frequency of genera *Blautia*, *Butyricimonas*, *Faecalibacterium*, *Lactococcus*, *Leuconostoc*, *Oscillospira*, *Propionibacterium*, and *Ruminococcus*, the presence of which is also associated with beneficial effects on the body, and their microbiota in IC samples was also reduced. Moreover, it should be noted that there were 8 genera detected in the same proportions as in the feces of MS patients: *Bradyrhizobium*, *Curvibacter*, *Methylobacterium*, *Sediminibacterium*, *Sphingomonas*, *Stenotrophomonas*, and *Variovorax* in the anaerobic consortium of patients with MS. These taxa are currently relatively poorly studied and we can only note that they provided an increase in biological diversity and may have been important as representatives of a large community of microorganisms inhabiting the intestine. More detailed information on the frequency of detection of all taxa at the genus level is presented in Tables S3 and S4 (Supplementary Materials).

**Figure 3:**
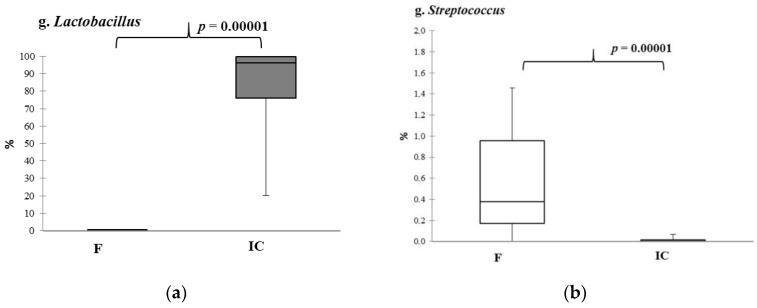
The relative abundance of *Lactobacillus* spp. (**a**) and *Streptococcus* spp. (**b**) in the fecal samples and indigenous consortiums. Notes: F—fecal samples; IC—indigenous consortium. Results are presented as median (25%; 75%).

An increase in representation only in the case *Lactobacillus* spp. (Figure 3a) and a decrease in relative abundance of *Streptococcus* spp. (Figure 3b) were obtained. The other genera were represented in a lower quantity compared to MS or not found at all (Table S2, Supplementary Materials).

### 3.2. Influence of the Indigenous Consortium Consumption on the Condition of Patients with MS and Their Microbiota

#### 3.2.1. Clinical and Laboratory Parameters before and after Therapy

##### Gastro Diaries

According to the results of gastro diaries while taking IC and after the end of the intake, 3 patients noted a significant improvement: the stool became regular, 1 time per day, according to the Bristol scale 3–4, imperative urge to defecate and tympanites disappeared, and general well-being improved. In total, 5 patients had irregular defecation when taking IC, previously the stool was regular (daily 1 time per day) but during 2 weeks after finishing the IC consumption the stool of all patients did not mark notable changes. Thus, the use of IC practically did not lead to any side effects. The dyspeptic changes that occurred were rare and quickly disappeared during the period of adaptation to a personalized product.

##### Eating Behavior

Eating disorders were revealed in all studied patients with MS. The questionnaire of eating behavior DEBQ in the groups revealed no differences (Supplementary Materials Table S1). According to the results of testing with the DEBQ questionnaire, patients scored the following points: block restrictive behavior—2.7 ± 0.2 points (norm < 2.4), emotional behavior 2.6 ± 0.2 points (norm < 1.8), and external behavior—4.9 ± 0.2 points (norm < 2.7). However, no statistically significant changes were found after taking IC.

##### Antropometria Results for Evaluation of Obesity Symptoms Study

**Figure 4:**
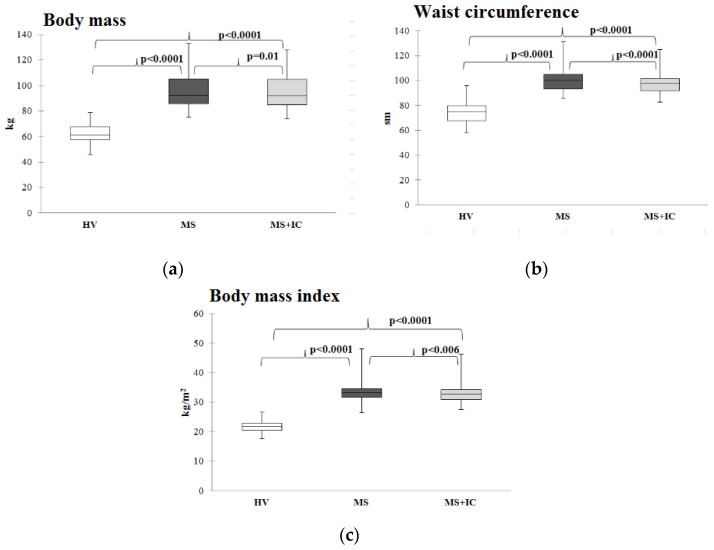
Body mass (**a**), waist circumference (**b**), and body mass index (**c**) of patients with MS before and after therapy. Notes: HV—healthy volunteers; MS—patients with metabolic syndrome; MS+IC—patients with metabolic syndrome after consumption of indigenous consortium. Results are presented as median (25%; 75%).

The body mass, waist circumference, and body mass index before and after therapy were higher in patients with MS than in the group of healthy volunteers, but these parameters decreased significantly after IC consumption (Figure 4).

**Figure 5:**
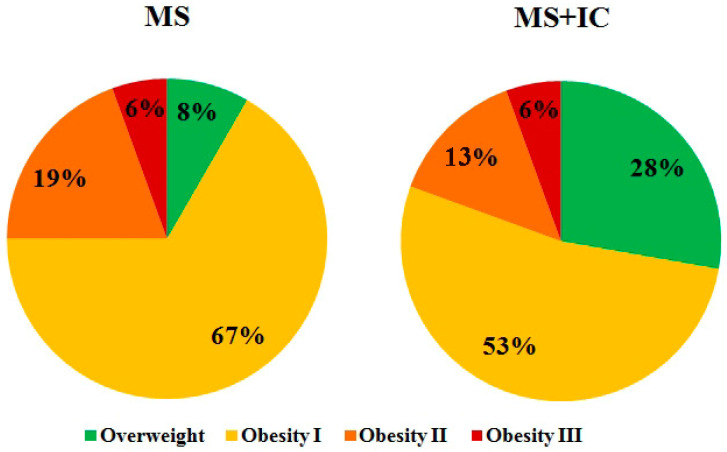
Distribution by severity of obesity before and after therapy. Notes: Classification of obesity by BMI: body weight deficit < 18.5 kg/m^2^; normal body weight 18.5–24.9 kg/m^2^; overweight 25–29.9 kg/m^2^; obesity of the 1st degree 30–34.9 kg/m^2^; obesity of the 2nd degree 35–39.9 kg/m^2^; obesity of the 3rd degree ≥ 40 kg/m^2^. MS—patients with metabolic syndrome; MS+IC—patients with metabolic syndrome after consumption of indigenous consortium.

As shown in Figure 5, in the proportion of overweight patients without obesity and grade 1 obesity after taking the consortium a tendency to decrease was noted (75% and 81%, respectively).

##### Biochemical Parameters

**Figure 6:**
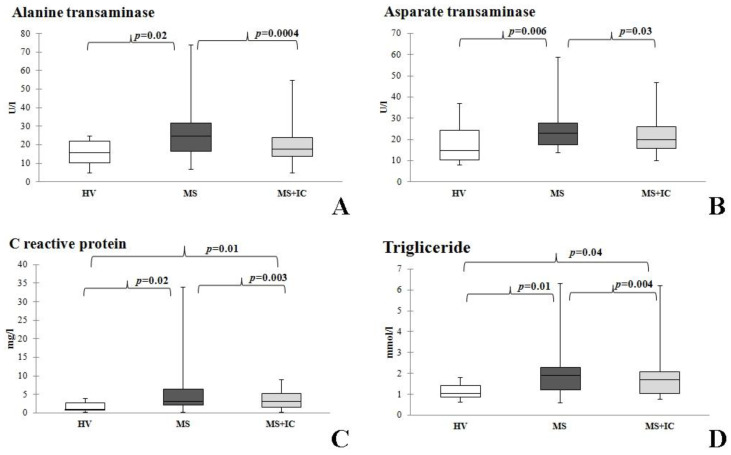
Biochemical parameters of blood serum before and after therapy. Alanine transaminase (**A**), Aspaparate transaminase (**B**), Creactive protein (**C**) and Trigliceride (**D**). Notes: HV—healthy volunteers; MS—patients with metabolic syndrome; MS + IC—patients with metabolic syndrome after consumption of indigenous consortium. Results are presented as median (25%; 75%).

The decrease in TG, liver enzymes (ALT, AST) level, and reduction in acute phase of inflammation protein (CRP) was revealed after IC usage (Figure 6). However, some of these (TG and CRP) remained higher compared to the group of healthy volunteers (Figure 6A,D).

#### 3.2.2. Microbiota Content before and after Therapy

##### Quantitative Polymerase Chain Reaction

**Figure 7:**
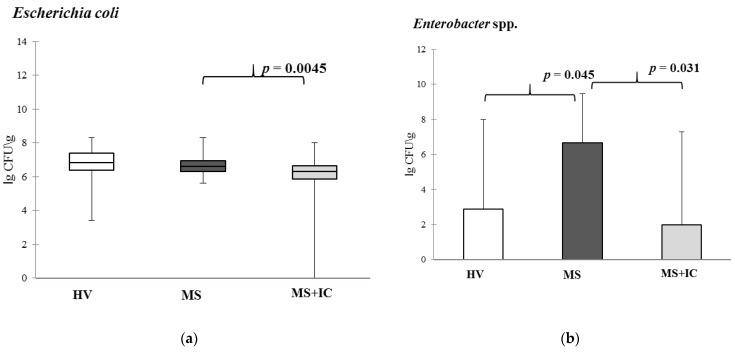

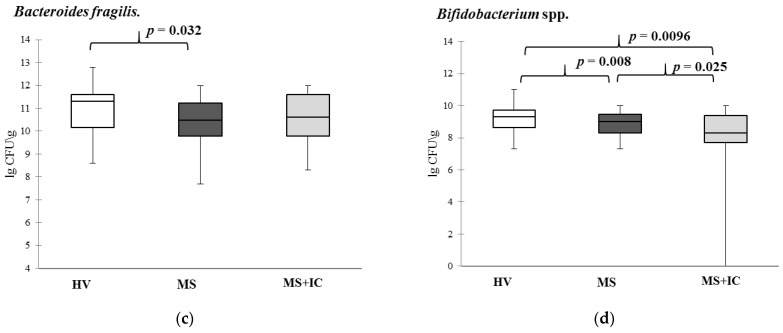
Microbiota content of patients with MS before and after IC by qPCR. *Escherichia coli* (**a**), *Enterobacter* spp. (**b**), *Bacteroides fragilis* (**c**) and *Bifidobacterium* spp. (**d**). Notes: HV—healthy volunteers; MS—patients with metabolic syndrome; MS+IC—patients with metabolic syndrome after consumption of indigenous consortium. Results are presented as median (25%; 75%).

The decrease in *Enterobacter* spp., *E. coli*, and *Bifidobacterium* spp. content and restoration (an increase in comparison with the control) quantity of *Bacteroides* spp. was revealed in intestinal microbiota (Figure 7).

##### Metagenome 16S rRNA Study

**Figure 8:**
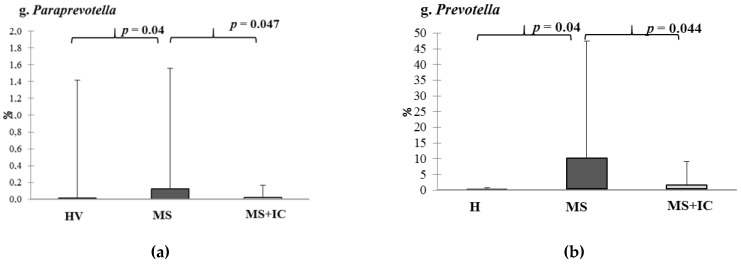

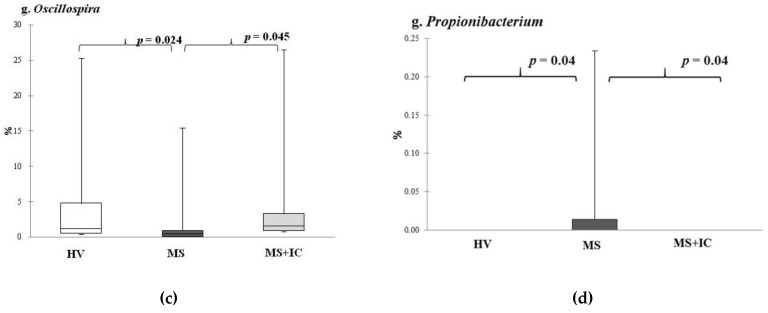
The relative abundance of microbiota content of patients with MS before and after IC by metagenome study (16S rRNA). Genera: *Paraprevotella* (**a**), *Prevotella* (**b**), *Oscillospira* (**c**) and *Propionibacterium* (**d**). Notes: HV—healthy volunteers; MS—patients with metabolic syndrome; MS + IC—patients with metabolic syndrome after consumption of indigenous consortium. Results are presented as median (25%; 75%).

In patients with MS alpha, the diversity did not change after IC consumption (Figure S3, Supplementary Materials). The relative abundances of genera *Paraprevotella* and *Prevotella* in feces of patients with MS were higher whereas *Propionibacterium* spp. and *Oscillospira* were lower than in fecal samples of healthy volunteers (Figure 8). All these parameters were normalized after IC consumption.

### 3.3. Summary Results and Correlation Analysis between Bacterial Taxa and Clinic and Laboratory Parameters

The summary effects of IC treatment on microbiome and clinic and laboratory parameters are presented in Table S5 (Supplementary Materials). In the correlation analysis, we focused on those bacterial taxa, whose quantity and representation changed after IC usage, as well as those clinic and laboratory parameters that are MS components and changed after treatment with IC.

**Figure 9:**
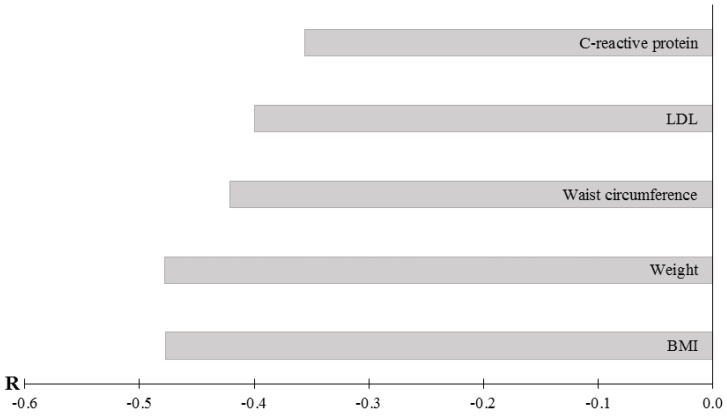
Correlation coefficients between *Bacteroides fragilis* content and anthropometric and biochemical parameters. Notes: total data for all patients, the results of the study are presented using *p* < 0.05. A search for correlations between the studied parameters was performed using Spearman’s test.

The negative correlations between *Bacteroides fragilis* quantitative content in fecal samples and anthropometric and serum blood parameters were revealed (Figure 9).

## 4. Discussion

The present paper is the first study devoted to the influence of the personified functional food product prepared by cultivation of indigenous fecal bacteria on the gut microbiota and clinical and laboratory parameters of patients with MS. Previously, we analyzed the effectiveness of IC on the animal experimental model [9].

A positive metabolic influence of microbiota employing the usage of bacteria as probiotics or by fecal microbiota transplantation is well documented in scientific literature [19]. The advantage of autoprobiotic microbial therapy is based on the usage for the indigenous bacteria which were adopted to the immune system of the host due the mechanisms of immune tolerance. This allows long conflict-free propagation of the bacteria in the gut in contrast to probiotics, which are usually rapidly eliminated from the gut [20]. Another advantage of autoprobiotics relative to the fecal microbiota transplantation is their safety profile and the ability to avoid the usage of bacteria with multiple antibiotic resistances or eukaryotic viruses that might be acquired from the donor [21]. Monostrain autoprobiotcs allowing the genome study of the bacteria used for microbial therapy seems the least dangerous but they are good for the recipients with relatively developed microbiota which need proper microbiota modulation. In the present study, we were evaluating another approach of making autoprobiotics based on fecal microbiota cultivation outside the host on selective media for several days. After 6 days of cultivation of the indigenous bacteria microbiota richness decreased to a certain degree but still it was represented by about 30 bacterial families with predominance of lactobacilli.

The study included patients with MS without serious disorders of carbohydrate metabolism. MS is usually manifested with abdominal type of obesity, violation of the lipid profile and arterial hypertension. Analysis of eating behavior of the group of patients under study revealed significant disorders, which could be a trigger for the development of severe MS associated with type II diabetes or cardio-vascular diseases and the course of the pathology being examined. These patients had elevated levels of ALT, AST, and triglycerides, which indicated liver dysfunction and lipid metabolism disorders. It attracted an increase in CRP which could indicate the presence of small inflammation, metabolic endotoxemia associated with an increase in the population of Gram-negative bacteria containing lipopolysaccharides (LPS), which might be a cause of metabolic endotoxemia [22].

As shown by comparative studies of the composition of fecal samples from patients with MS and the consortiums of bacteria obtained during their cultivation, the selected cultivation conditions allow for selection of bacteria strains, as a result of which mainly beneficial bacteria, belonging to the genera of *Lactobacilli*, *Enterococci*, and *Bifidobacteria* used to create probiotics can be accumulated. At the same time, a number of opportunistic Gram-negative bacteria *Campylobacter*, *Citrobacter*, *Desulfovibrio*, *Haemophilus*, *Bilophila*, *Paraprevotella*, *Prevotella*, and *Burkholderia* disappear from samples or their number and representation decreases. The presence of these LPS in the fecal samples of patients could be one of the reasons for the development of the so-called minor inflammation that accompanies MS and was confirmed by an increase in the level of CRP. The approach which favors mainly lactic acid bacteria allows to abolish most of intestinal pathogens and eukaryotic virus contamination. The introduction of IC practically did not cause significant changes in digestive functions (frequency and characteristics of stool, dyspeptic symptoms) and did not affect the eating behavior of patients.

The administration of this IC in patients with MS caused a significant health effect, which was reflected in weight loss, improvement in biochemical parameters of the blood serum, and decrease in symptoms of the low grade inflammation.

The positive changes in anthropometric and biochemical parameters obtained in our work correlate with several previous studies devoted to the effect of probiotics or synbiotics on the patients with MS. A randomized placebo-controlled study evaluating the effect of a two-month intake of a synbiotic (including *Lactobacillus plantarum* PBS067, *Lactobacillus acidophilus* PBS066, and *Lactobacillus reuteri* PBS072 with a prebiotic) showed a significant effect of taking a synbiotic on carbohydrate, lipid metabolism, and obesity criteria. In the group of patients treated with the synbiotic, there was a statistically significant decrease in weight coefficient, insulin levels, total cholesterol, HDL cholesterol, non-HDL cholesterol, fasting plasma triglycerides, as well as a decrease in the value of the visceral obesity index. There was also a significant decrease in blood pressure (BP) and fasting plasma glucose levels in the synbiotic group [23].

In the other randomized study of 46 obese patients treated with a synbiotic containing Lactobacillus casei, Lactobacillus rhamnosus, Streptococcus thermophilus, Bifidobacterium breve, Lactobacillus acidophilus, Bifidobacterium longum, Lactobacillus bulgaricus, and fructo-oligosaccharides), and following a low-calorie diet, the positive effect of taking the synbiotic was also demonstrated. There was a greater decrease in body weight, BMI, fasting plasma glucose, insulin concentration, and insulin resistance index (HOMA-IR) in the synbiotic group compared to the placebo group. At the same time, the level of glucagon-like peptide-1 and tyrosine–tyrosine peptide, which suppress appetite and promotes weight loss, increased when taking the synbiotic [24].

The partial compensation of lipid profile disorders revealed by us in this work after the use of IC was manifested in a decrease in the level of triglycerides in the blood serum. Previously, such changes were noted after the introduction of probiotic lactobacilli [7].

The elevated levels of liver enzymes in the blood sera of patients with MS prior to IC administration most likely reflected formation of metabolic-associated liver disease (MAFLD) in this group of patients. We believe that decrease in ALT and AST levels after taking IC may be associated with a decrease in Gram-negative bacteria (*Enterobacter* spp., *Escherichia coli*) quantity. Another group of researchers evaluating the association of MAFLD with changes in the gut microbiota composition in 52 obese patients found more *Enterobacteriaceae* and *Escherichia coli* in patients with greater severity of MAFLD associated with higher levels of AST [25]. Similar data were obtained in 2017 in the study comparing the gut microbiota of patients with MAFLD and healthy individuals: more severe fibrosis in MAFLD was associated with a greater number of *Enterobacteriaceae* and *Escherichia*/*Shigella bacteria*.

A relative increase in the representation of the genus *Oscillospira* after IC consumption might also have contributed to the positive effect of IC administration. Bioinformatic analysis demonstrated that the abundance of this taxon in the intestinal microbiota was in strong association between representation of this bacteria and diseases associated with obesity-related chronic inflammatory and metabolic diseases. Some studies confirm the inverse correlation of this bacteria genus with body weight [26]. Oscillospira species from the *Clostridial* cluster belonging to the *Lachnospiraceae* family are butyrate producers, and at least some of them have the ability to utilize glucuronate, a common animal-derived sugar. The presence of this genus is reduced in diseases that involve inflammation [27]. In addition, several studies have confirmed that *Oscillospira* spp. is strongly associated with leanness or lower BMI in children and adults [28,29].

The positive effects of *Oscillospira* in obesity-related s down by other microbes. These bacteria also produce fatty acid as a byproduct of carbohydrate fermentation which can then be used as energy by the host. It was discovered that Cytochrome *bd* oxidase is essential to oxygen consumption. This can allow other obligate anaerobes to survive in the now oxygen-reduced microenvironment [30,31]. Polysaccharide A or outer membrane vesicles from nontoxigenic *B. fragilis* may mediate beneficial interactions with the host [32]. The intestinal microbial competition of *B. fragilis* is supported by two pervasive ecological drivers: non-contact-dependent secretory antimicrobial proteins and the contact-dependent Type VI secretion system (T6SSs). The first known *Bacteroidales* secreted antimicrobial protein-1 (BSAP-1) is released in *B. fragilis* OMVs, which contain membrane attack/perforin (MACPF) domains that are used to lyse other bacterial cells or infect host cells via pore formation. BSAP-1 is an important competitive factor that affects the composition of the human intestinal microbiota [33]. Stimulation of innate immunity, antibacterial activity due to bacteriocins, creation of a favorable microenvironment for beneficial microbes, and neutralization of LPS make it possible to consider it as a potential genus for searching for a probiotic strain. This is confirmed in our work by a negative correlation with the majority of disorders identified in the group of patients with MS under consideration.

It is necessary to note the potential influence of lactobacilli, the leading taxon in IC, whose antibacterial immunomodulatory anti-inflammatory effect is described in the literature but was overshadowed in this study. Changes in the composition, including species composition, are beyond the scope of this work.

## 5. Conclusions

Patients with metabolic syndrome consuming the functional food made out of the indigenous consortium of bacteria cultivated on the artificial media demonstrated a significant improvement in several clinical and laboratory parameters such as BMI, body weight, CRP, ALT, and AST. Intestinal microbiota analysis demonstrated a reduction in the numbers of *Proteobacteria* phylum and an increase in the abundance of *Bacteroides fragilis* and *Oscillospira* spp. All these allow us to consider that the complex of co-grown indigenous bacteria with a predominance of lactobacilli may be suggested as a component of the comprehensive therapy of the diseases associated with the so-called “small inflammation” such as MS.

## Figures and Tables

**Table 1:** Frequency of different taxa identification in the composition of fecal samples and the indigenous consortium by qPCR study.

Genera in Which Frequency of Identification Was Increased	Genera of Opportunistic Bacteria in Which Frequency of Identification Was Decreased
*Agrobacterium*, *Bacillus* *Enterococcus*, *Lactobacillus*, *Pediococcus*	*Campylobacter*, *Citrobacter*, *Desulfovibrio*, *Haemophilus*, *Bilophila*, *Paraprevotella*, *Prevotella*, *Steptococcus*, *Granulicatella Burkholderia*, *Pseudomonas*, *Veillonella*, *Propionibacterium*

## Data Availability

Data available in a publicly accessible repository.

## Supplementary Materials

The following supporting information can be downloaded at: https://www.mdpi.com/article/10.3390/microorganisms10081574/s1, Figure S1: Gender ratio (a) and age (b) of patients with MS; Figure S2. Alfa-diversity of fecal samples and indigenous consortium; Figure S3. Alfa-diversity Index: Shannon of fecal samples of metabolic syndrome (MS), metabolic syndrome after after taking indigenous consortium (MS+IC) and healthy people (V); Table S1. Study design; Table S2. Frequency of occurrence of microbiota representatives in F and IC determined by qPCR using the kit; Table S3. Frequency of occurrence of microbiota representatives in F and IC determined by qPCR; Table S4. Frequency of occurrence of microbiota representatives in F and IC determined by metagenome 16S rRNA; Table S5. Summary results.

## References

[1] Eckel R.H., Grundy S.M., Zimmet P.Z. (2005). The metabolic syndrome. Lancet.

[2] Laaksonen D.E., Niskanen L., Lakka H.M., Lakka T.A., Uusitupa M. (2004). Epidemiology and treatment of the metabolic syndrome. Ann. Med..

[3] Kurmangulov A.A., Dorodneva E.F., Isakova D.N. (2016). Functional activity of intestinal microbiota with metabolic syndrome. Obes. Metab..

[4] Buttó L., Haller D. (2016). Dysbiosis in intestinal inflammation: Cause or consequence. Int. J. Med. Microbiol..

[5] Wang P.X., Deng X.R., Zhang C.H., Yuan H.J. (2020). Gut microbiota and metabolic syndrome. Chin. Med. J..

[6] Santini A., Novellino E. (2018). Nutraceuticals-shedding light on the grey area between pharmaceuticals and food. Expert Rev. Clin. Pharmacol..

[7] Festi D., Schiumerini R., Eusebi L.H., Marasco G., Taddia M., Colecchia A. (2014). Gut microbiota and metabolic syndrome. World J. Gastroenterol..

[8] Solov’eva O.I., Simanenkov V.I., Suvorov A.N., Ermolenko E.I., Shumihina I.A., Svirido D.A. (2017). The use of probiotics and autoprobiotics in the treatment of irritable bowel syndrome. Clin. Exp. Gastroenterol..

[9] Suvorov A., Karaseva A., Kotyleva M., Kondratenko Y., Lavrenova N., Korobeynikov A., Kozyrev P., Kramskaya T., Leontieva G., Kudryavtsev I. (2018). Autoprobiotics as an approach for restoration of personalised microbiota. Front. Microbiol..

[10] Gromova L.V., Ermolenko E.I., Sepp A.L., Dmitrieva Y.V., Alekseeva A.S., Lavrenova N.S., Kotyleva M.P., Kramskaya T.A., Karaseva A.B., Suvorov A.N. (2021). Gut Digestive Function and Microbiome after Correction of Experimental Dysbiosis in Rats by Indigenous Bifidobacteria. Microorganisms.

[11] Suvorov A., Simanenkov V., Gromova L., Kolodjieva V., Tsapieva A., Chernish A., Solovieva O., Ermolenko E., Ivanova I. (2011). Enterococci as probiotics or autoprobiotics. Prebiotics and Probiotics Potential for Human Health.

[12] de Groot P.F., Frissen M.N., de Clercq N.C., Nieuwdorp M. (2017). Fecal microbiota transplantation in metabolic syndrome: History, present and future. Gut Microbes.

[13] Kahn S.A., Gorawara-Bhat R., Rubin D.T. (2012). Fecal bacteriotherapy for ulcerative colitis: Patients are ready, are we?. Inflamm. Bowel Dis..

[14] World Medical Association (2013). World Medical Association Declaration of Helsinki: Ethical principles for medical research involving human subjects. JAMA.

[15] Li W., Chang Y. (2017). CD-HIT-OTU-MiSeq, an improved approach for clustering and analyzing paired end MiSeq 16S rRNA sequences. BioRxiv.

[16] DeSantis T., Hugenholtz P., Larsen N., Rojas M., Brodie E., Keller K., Huber T., Dalevi D., Hu P., Andersen G. (2006). Greengenes, a chimera-checked 16S rRNA gene database and workbench compatible with ARB. Appl. Environ. Microbiol..

[17] van Strien T., Frijters J.E.R., Bergers G.P.A., Defares P.B. (1986). The Dutch Eating Behavior Questionnaire (DEBQ) for assessment of restrained, emotional, and external eating behavior. Int. J. Eat. Disord..

[18] Simanenkov V., Tikhonov S., Lischuk N. (2017). Treatment compliance at initial and maintenance therapy at gastroesophageal reflux disease. Russ. J. Gastroenterol. Hepatol. Coloproctol..

[19] Yu E.W., Gao L., Stastka P., Cheney M.C., Mahabamunuge J., Torres Soto M., Ford C.B., Bryant J.A., Henn M.R., Hohmann E.L. (2020). Fecal microbiota transplantation for the improvement of metabolism in obesity: The FMT-TRIM double-blind placebo-controlled pilot trial. PLoS Med..

[20] Suez J., Zmora N., Segal E., Elinav E. (2019). The pros, cons, and many unknowns of probiotics. Nat. Med..

[21] Park S.Y., Seo G.S. (2021). Fecal Microbiota Transplantation: Is It Safe?. Clin. Endosc..

[22] Cani P.D., Possemiers S., Van De Wiele T., Guiot Y., Everard A., Rottier O., Geurts L., Naslain D., Neyrinck A., Lambert D.M. (2009). Changes in gut microbiota control inflammation in obese mice through a mechanism involving GLP-2-driven improvement of gut permeability. Gut.

[23] Cicero A.F.G., Fogacci F., Bove M., Giovannini M., Borghi C. (2021). Impact of a short-term synbiotic supplementation on metabolic syndrome and systemic inflammation in elderly patients: A randomized placebo-controlled clinical trial. Eur. J. Nutr..

[24] Rabiei S., Hedayati M., Rashidkhani B., Saadat N., Shakerhossini R. (2019). The Effects of Synbiotic Supplementation on Body Mass Index, Metabolic and Inflammatory Biomarkers, and Appetite in Patients with Metabolic Syndrome: A Triple-Blind Randomized Controlled Trial. J. Diet. Suppl..

[25] Lanthier N., Rodriguez J., Nachit M., Hiel S., Trefois P., Neyrinck A.M., Cani P.D., Bindels L.B., Thissen J.P., Delzenne N.M. (2021). Microbiota analysis and transient elastography reveal new extra-hepatic components of liver steatosis and fibrosis in obese patients. Sci. Rep..

[26] Garcia-Mantrana I., Selma-Royo M., Alcantara C., Collado M.C. (2018). Shifts on gut microbiota associated to mediterranean diet adherence and specific dietary intakes on general adult population. Front. Microbiol..

[27] Gophna U., Konikoff T., Nielsen H.B. (2017). Oscillospira and related bacteria—From metagenomic species to metabolic features. Environ. Microbiol..

[28] Konikoff T., Gophna U. (2016). Oscillospira: A central, enigmatic component of the human gut microbiota. Trends Microbiol..

[29] Beaumont M., Goodrich J.K., Jackson M.A., Yet I., Davenport E.R., Vieira-Silva S., Debelius J., Pallister T., Mangino M., Raes J. (2016). Heritable components of the human fecal microbiome are associated with visceral fat. Genome Biol..

[30] Yang J., Li Y., Wen Z., Liu W., Meng L., Huang H. (2021). Oscillospira—A candidate for the next-generation probiotics. Gut Microbes.

[31] Wexler H.M. (2007). Bacteroides: The good, the bad, and the nitty-gritty. Clin. Microbiol. Rev..

[32] Sun F., Zhang Q., Zhao J., Zhang H., Zhai Q., Chen W. (2019). A potential species of next-generation probiotics? The dark and light sides of Bacteroides fragilis in health. Food Res. Int..

[33] Chatzidaki-Livanis M., Coyne M.J., Comstock L.E. (2014). An antimicrobial protein of the gut symbiont Bacteroides fragilis with a MACPF domain of host immune proteins. Mol. Microbiol..

