# Supplementary material for: Consortium of Indigenous Fecal Bacteria in the Treatment of Metabolic Syndrome
Source: Microorganisms. 2022 Aug 5;10(8):1574. doi: 10.3390/microorganisms10081574 (PMC9414820; doi:10.3390/microorganisms10081574)
Supplement: Supplementary file 1 [file microorganisms-10-01574-s001.zip › microorganisms-1820561-supplementary.pdf]

SUPPLEMENTARY MATERIALS

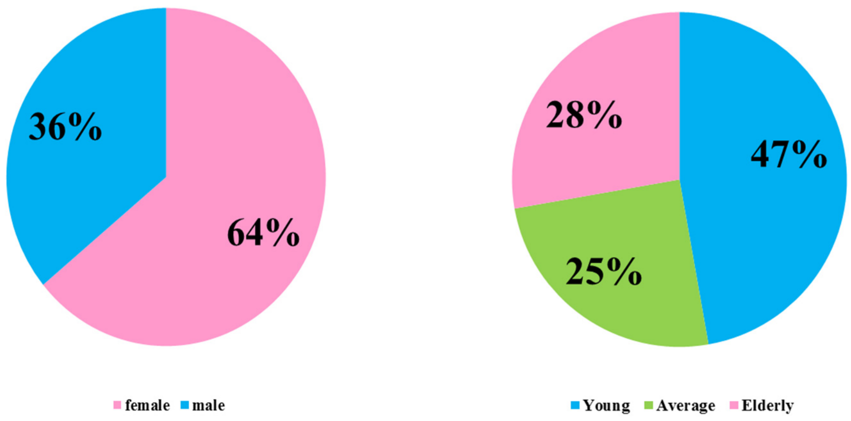

(a) (b)

Figure S1. Gender ratio (a) and age (b) of patients with MS.
Notes: Classification of ages according to WHO 2021: age up to 44 years - young; 45-59 – average; 60-74 – elderly; 75-89 – senile; 90-100 and older – centenarians.

Table S1. Study design.

	**Quantity of patients**	**0-10 days**	**15 day**	**16-25 days**	**26-28 days**
Total MS	44	Patients and healthy volunteers selection	IC making	Treatment	
MS	36	Blood, Fecal samples, antropometry, clinical, biochemical study	IC making	-	-
MS+IC	36			Soy milk fermented personified product, gastro scale	Blood, fecal samples

Table S2. Frequency of occurrence of microbiota representatives in F and IC determined by qPCR using the kit

**Genera**	**Frequency of occurrence of taxa representatives**		
	Fecal samples	IC	**$\chi 2$ test with Yates correction, *p* =**
Bifidobacterium spp.	100.00%	31.03%	0.00001
Bacteroides fragilis	97.06%	37.93%	0.00001
Faecalibacterium prausnitzii	97.06%	20.69%	0.00001
Bacteroides thetaomicron	38.24%	0.00%	0.0006
Akkermansia muciniphila	55.88%	0.00%	0.00001
Enterobacter spp.	38.24%	10.34%	0.0248

Table S3. Frequency of occurrence of microbiota representatives in F and IC determined by qPCR .

Genera	Frequency of occurrence of taxa representatives Lg CFU/g > 5		
	Fecal samples	IC	**χ2 test with Yates correction, *p*=**
Lactobacillus spp.	35.29%	93.10%	0.00001
Enterococcus spp.	0.00%	27.59%	0.0038

Table S4. Frequency of occurrence of microbiota representatives in F and IC determined by metagenome 16S rRNA.

Genera	Frequency of occurrence of taxa representatives		
	Fecal samples	IC	**χ2 test with Yates correction, *p*=**
Acidaminococcus	7.69%	0.00%	1.0000
Acinetobacter	15.38%	0.00%	0,4617
Actinomyces	46.15%	0.00%	0.0200
Aggregatibacter	15.38%	0.00%	0.4617
Agrobacterium	0.00%	7.69%	1.0000
Akkermansia	46.15%	0.00%	0.0200
Anaerostipes	23.08%	0.00%	0.2196
Atopobium	46.15%	0.00%	0.0200
Bacillus	0.00%	7.69%	1.0000
Bacteroides	84.62%	0.00%	0.0001
Bifidobacterium	53.85%	7.69%	0.0336
Bilophila	46.15%	0.00%	0.0200
Blautia	61.54%	0.00%	0.0029
Bradyrhizobium	46.15%	53.85%	1.0000
Bulleidia	53.85%	0.00%	0.0080
Burkholderia	23.08%	15.38%	1.0000
Butyricimonas	30.77%	0.00%	0.1030
Campylobacter	7.69%	0.00%	1.0000
Catenibacterium	38.46%	0.00%	0.0465
Caulobacter	38.46%	46.15%	1.0000
Christensenella	7.69%	0.00%	1.0000
Citrobacter	7.69%	0.00%	1.0000
Clostridium	84.62%	0.00%	0.0001
Collinsella	53.85%	0.00%	0.0080
Coprococcus	53.85%	0.00%	0.0080
Corynebacterium	15.38%	0.00%	0.4617
Cupriavidus	7.69%	0.00%	1.0000
Curvibacter	15.38%	7.69%	1.0000
Desulfovibrio	15.38%	0.00%	0.4617
Dialister	38.46%	0.00%	0.0465
Dorea	84.62%	0.00%	0.0001
Eggerthella	23.08%	0.00%	0.2196

Enterococcus	0.00%	30.77%	0.1030
Exiguobacterium	7.69%	0,00%	1.0000
Faecalibacterium	92.31%	0.00%	0,00001
Fimbriimonas	15.38%	0.00%	0.4617
Gemella	23.08%	0.00%	0.2196
Granulicatella	61.54%	38.46%	0.4328
Haemophilus	15.38%	0.00%	0.4617
Lachnospira	46,15%	0.00%	0.0200
Lactobacillus	61.54%	100.00%	0.0465
Lactococcus	23.08%	0.00%	0.2196
Leuconostoc	15.38%	0.00%	0.4617
Megamonas	15.38%	0.00%	0.4617
Megasphaera	23.08%	0.00%	0.2196
Nevskia	7.69%	0.00%	1.0000
Ochrobactrum	7.69%	0.00%	1.0000
Odoribacter	23.08%	0.00%	0.2196
Oribacterium	7.69%	0.00%	1.0000
Oscillospira	92.31%	7.69%	0.0001
Parabacteroides	69.23%	0.00%	0.0010
Paraprevotella	38.46%	0.00%	0.0465
Parvimonas	7.69%	0.00%	1.0000
Pediococcus	15.38%	38.46%	0.3766
Phascolarctobacterium	38.46%	0.00%	0.0465
Polaromonas	30.77%	15.38%	0.6416
Prevotella	69.23%	0.00%	0.0010
Propionibacterium	30.77%	7.69%	0.3196
Pseudomonas	30.77%	23.08%	1.0000
Psychrobacter	0.00%	7.69%	1.0000
Rothia	7.69%	0.00%	1.0000
Ruminococcus	84.62%	0.00%	0.0001
Scardovia	7.69%	0.00%	1.0000
Sediminibacterium	61.54%	69.23%	1.0000
Selenomonas	7.69%	0.00%	1.0000
Serratia	23.08%	0.00%	0.2196
Slackia	46.15%	0.00%	0.0200
Sphingomonas	76.92%	84.62%	1.0000
Staphylococcus	7.69%	23.08%	0.5867
Sutterella	38.46%	0.00%	0.0465
Turicibacter	38.46%	0.00%	0.0465
Veillonella	53.85%	0.00%	0.0080

Table S5. Summary results.

	Changes after IC therapy	comparison with a group HV
**Anthropometry parmeters**		
Body Mass	↓	>
Waist circumference	↓	>
Body mass index	↓	>
**Biochemical parameters**		
Triglyceride	↓	>
C reactive protein	↓	>
Alanine aminotranferase	↓	ND
Aspartate aminotransferase	↓	ND
**Quantitative content**		
***Enterobacter* spp.**	↓	ND
Esherichia coli	↓	ND
Bifidobacterium spp.	↓	<
Bacteroides fragilis	**↑ tendency**	ND
**Relative abundance**		
Prevotella spp.	↓	ND
Paraprevotella spp.	↓	ND
Oscillospira spp.	↑	ND
Propionibacterium spp.	↓	ND

Notes: ND – no differences.

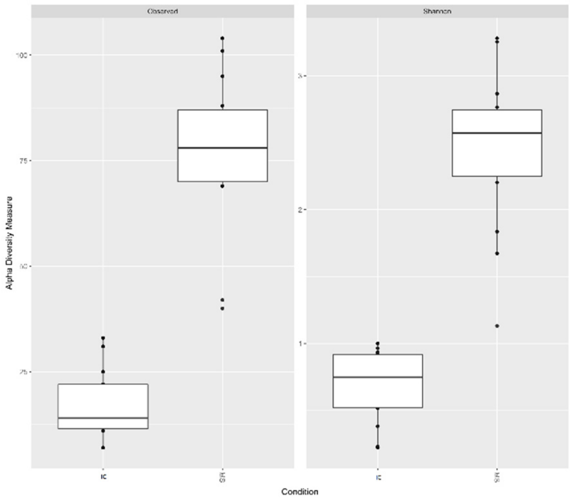

Figure S2. Alfa-diversity of fecal samples and indigenous consortium.

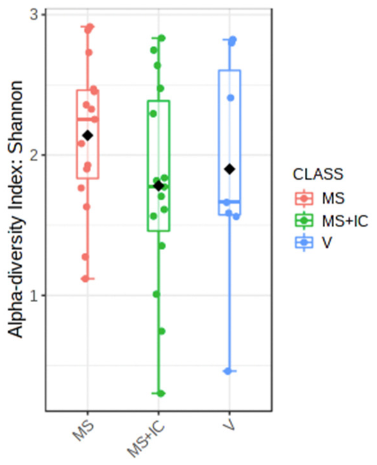

Figure S3. Alfa-diversity Index: Shannon of fecal samples of metabolic syndrome (MS), metabolic syndrome after after taking indigenous consortium (MS+IC) and healthy people (V).
